# Importance of Zinc Nanoparticles for the Intestinal Microbiome of Weaned Piglets

**DOI:** 10.3389/fvets.2022.852085

**Published:** 2022-06-02

**Authors:** Daria Baholet, Sylvie Skalickova, Andrej Batik, Svetlana Malyugina, Jiri Skladanka, Pavel Horky

**Affiliations:** ^1^Department of Animal Nutrition and Forage Production, Mendel University in Brno, Brno, Czechia; ^2^Department of Animal Morphology, Physiology and Genetics, Mendel University in Brno, Brno, Czechia

**Keywords:** piglets' weaning, diarrhea, microbiome, zinc oxide, nanotechnology, antimicrobial effects, antibiotic replacement, gastro-intestinal tract

## Abstract

The scientific community is closely monitoring the replacement of antibiotics with doses of ZnO in weaned piglets. Since 2022, the use of zinc in medical doses has been banned in the European Union. Therefore, pig farmers are looking for other solutions. Some studies have suggested that zinc nanoparticles might replace ZnO for the prevention of diarrhea in weaning piglets. Like ZnO, zinc nanoparticles are effective against pathogenic microorganisms, e.g., *Enterobacteriaceae* family *in vitro* and *in vivo*. However, the effect on probiotic *Lactobacillaceae* appears to differ for ZnO and zinc nanoparticles. While ZnO increases their numbers, zinc nanoparticles act in the opposite way. These phenomena have been also confirmed by *in vitro* studies that reported a strong antimicrobial effect of zinc nanoparticles against *Lactobacillales* order. Contradictory evidence makes this topic still controversial, however. In addition, zinc nanoparticles vary in their morphology and properties based on the method of their synthesis. This makes it difficult to understand the effect of zinc nanoparticles on the intestinal microbiome. This review is aimed at clarifying many circumstances that may affect the action of nanoparticles on the weaning piglets' microbiome, including a comprehensive overview of the zinc nanoparticles *in vitro* effects on bacterial species occurring in the digestive tract of weaned piglets.

## Introduction

ZnO has been used as antibacterial agent in conventional monogastric breeding zootechnical systems for many years (1). It has been frequently applied during piglets' weaning which is accompanied by oxidative stress, barrier disfunction, and disturbance of intestinal microflora, which then can be responsible for villi atrophy, growth retardation, and diarrhea (2). The pharmacological dose of dietary Zn (2,000–3,000 mg/kg of Zn in diet) has been widely accepted because of its effective function in promoting growth and alleviating the diarrhea of weaned piglets (3). However, when high levels of Zn are consumed by pigs, significant portions of the Zn are excreted in urine and feces. Thus, it raises concerns about environmental pollution (4) and leads to a negative perception of ZnO by the public (5). Environmental concerns relate mainly to run-off of Zn to surface water and groundwater. Sandy soils are the most susceptible to these processes. The accumulation of Zn in topsoil is another concern. Accumulated Zn levels may be as high as the concentrations that are potentially toxic for organisms living there (6).

Moreover, the extensive application of high doses of dietary Zn may affect the digestibility and stability of other trace elements (7). To the contrary, ZnO can be used for improving barrier function, regulating the oxidative state, modulating the immune response, and altering the intestinal microbiota (8).

Recently, several modern methods, such as bioengineering, biotechnology, genetic engineering, and nanotechnology have aided animal production. Nanotechnology has revolutionized the commercial use of nano-sized minerals in medicine, food and biotechnology, pharmaceutical applications, and more (1). The International Organization for Standardization (ISO) has described a nanomaterial as a “material with any external nanoscale dimension or having the internal nanoscale surface structure” (2010). The EU Commission defines the term nanomaterial as a manufactured or natural material that possesses unbound, aggregated, or agglomerated particles where external dimensions are within the 1–100 nm size range (9).

Several types of nanoparticles (NPs) are used in animal science due to their high antibacterial activity, chemical stability, and solubility (10). Various studies have proved that the efficacy and bioactive properties of nanomaterials strongly depend on the surface, chemical, and structural composition (11). There is no doubt that the emergence of nanotechnology has opened new options that allow us to cope with bacterial infections and resistance. This is proven by an increase in the number of studies on weaning piglets treated by ZnNPs. The antimicrobial activity of ZnNPs is enabled by their small size and high relative surface area (10) ZnNPs are more active against gram-positive bacteria than other NPs of the same group of elements (10). For this reason, ZnNPs are the third-highest globally produced nanometals (1).

If ZnNPs have a truly positive impact in the field of pig production, their use could bring economic benefits to pig breeding. In contrast, their physico-chemical properties, fate and effect on the animals' physiology, and gastrointestinal microbiota must be carefully considered. This review summarizes the available data of ZnNPs' effects against gastro-intestinal microbiota and its impact on the occurrence of post-weaning diarrhea.

## Importance of Zn in Pigs Organism

Zn is an essential trace element that is essential in protein synthesis and proper physiological functions of body, weight gain (12) and reproduction (1). However, Zn has a strong toxic potential when the concentration within a biological system exceeds a certain limit. Therefore, the regulation of Zn uptake, redistribution, and excretion within an organism must be tightly controlled (13). Adequate Zn supplementation is important to prevent any immune system alteration, decrease susceptibility to bacterial infections, and to maintain the integrity of the intestinal tight junctions (14, 15). Many organs may be affected by Zn deficiency, especially the immune system, which is extremely vulnerable to changes in Zn levels. Almost every immunological event is somehow influenced by Zn, which has been comprehensively described in the review by Maares and Haase (7). For example, polymorphonuclear cells are first responders of innate immunity. It has been shown that their chemotaxis and phagocytosis are reduced in Zn deficiency (16). Another example can be made in the event of phagocytosis, where pathogens are destroyed by the activity of NADPH oxidases, which have been shown to be inhibited by both Zn deficiency and excess Zn (17). With respect to immunologic studies in gastro-intestinal tract (GIT), the modulatory effect on intestinal inflammation was confirmed by several *in vivo* studies on piglets (8, 18) supplemented with ZnO, reporting the decrease of inflammation markers; IFN-γ, TNF-α, and IL-6.

On the molecular level, Zn reactivity enables it to be a part of the catalytic cofactor of many specific peptides and proteins (metalloproteins) involved in cellular processes, such as DNA synthesis, RNA-transcription, and cell division (19, 20). Zn is required for the healthy condition of the epidermis, epithelium, skin, and hooves (1). Last but not least, Zn has several antioxidant effects. These comprise, for example, Zn as a cofactor of the Cu/Zn superoxide dismutase that catalyzes the dismutation of the superoxide radical (O_2_) into the less harmful O2 and H_2_O_2_, which is then detoxified by catalase and glutathion peroxidase. Zn also inhibits NADPH oxidases, resulting in reduced formation of reacive oxygen species (ROS) (21). Several systematic reviews of Zn's role in the mammalian organism have been undertaken (22–24). However, new findings in this field are still developing. This underlines the importance and complexity of Zn in the body.

### Zn Bioavailability and Impact on GIT

Zn from dietary intake is mostly absorbed in the small intestine, with the main absorption taking place in the duodenum followed by the proximal jejunum. Absorption occurs primarily through apical transport with the participation of Zn transporters in the apical membrane (25). There are two major protein families which include mammalian Zn transporters. The first group of transporters are ZIP (Zrt/Irt-like proteins), which are responsible for transporting Zn into the cytosol from either the extracellular space or from intracellular compartments. The second group of Zn transporters (SLC30 A1 - A10) transport Zn from the cytosol to the extracellular space or to intracellular organelles such as znosomes. Znosomes are vesicles able to bind large amounts of Zn (16). Metallothionein, one of the Zn binding proteins responds very rapidly to Zn status in the body (26). Up to 20% of intracellular Zn is bound to metallothionen and can be rapidly released. It has been shown that the high dietary levels of Zn lead to an increase of intestinal metallothionein synthesis, Zn sequestration, and altered binding capacity. Therefore, it appears that metallothionein may have a role in both Zn storage and regulation (7). Monitoring of metallothionein and metal transporters gene expression appears to be a promising additional marker for mineral status, mineral excretion, or antioxidant capacity in the field of pig breeding. A recently published study has shown that the inadequate dose of Zn and the antagonistic effect of other minerals can influence digestive enzyme activity and metal transporter gene expression (27).

The bioavailability of Zn depends on the composition of the food or feed. Indigestible plant ligands such as phytate, dietary fibers and lignin, can chelate Zn and inhibit its absorption. Other factors that affect the absorption of Zn are calcium and iron (16). Case et al. reported that the bioavailability of Zn from ZnO is lower than from other sources, such as ZnSO_4_, Zn-methionine, and Zn-lysine. The unused ZnO in pigs' GIT might affect the overall mineral absorption (due to an antagonistic effect) and it is then excreted in the feces (12). The high amount of Zn in the slurry leads to a significant accumulation of Zn in the environment, which appears to be the biggest concern facing its application. Despite this, excess of inorganic Zn added to the diet still meets the Zn requirement for growth performance of animals (14). The deficiency of Zn may lead to severe disorders such as eye and skin lesions, hair loss, delayed sexual maturity. Weaned piglets develop first signs of clinical Zn deficiency (feed refusal) after ~10 days of low Zn intake (13). Dietary recommendations for pigs range ~80–150 mg/kg of diet (18).

In pig breeding practice, high doses of Zn have been used as an effective antibiotic replacement to prevent diarrhea in weaned piglets. The most frequently administered dose is Zn 2,000–3,000 mg/kg of diet for such purposes. Numerous studies have been conducted to clarify the antimicrobial effect on ZnO. However, the exact mechanism of Zn is not completely known, and individual results are still inconsistent. Due to the fact that a high dose of Zn is not fully absorbed into the body, a large part passes to the GIT, where the following mechanisms take place: (i) a mucosal layer formed on the intestinal epithelium, which prevents adhesion of pathogenic microorganisms; (ii) ratio of villi and crypts increases; (iii) high doses of Zn in an alkaline environment lyse the cytoplasmic membranes of microorganisms; (iv) Zn reduces ion secretion into the intestinal lumens thereby increasing water resorption and diarrhea prevention; (v) paracellular permeability reduces and thus prevents translocation of pathogenic microorganisms; (vi) free Zn ions can promote oxidation-reduction interactions (18, 28–30). Nevertheless, these mechanisms are likely to complement each other (28). Zn medication doses are given to weaned piglets for a maximum of 2 weeks after weaning. If the administration time were longer, the microbiome could be severely disrupted in terms of reducing lactobacilli as well as cellulolytic and proteolytic bacteria. Moreover, the disorders of fiber and protein digestion may occur (31). As a result, the animals' growth capacity would be reduced and fattening would be prolonged (32).

## ZnNPs as a Prospective Antimicrobial Agent

Emerging utilization of NPs in many industrial fields (including agriculture) has impacted the development of large-scale NPs production. Nanomaterials can be synthesized by a several methods such as chemical synthesis (bottom-up approach) or spray pyrolysis, thermal decomposition, molecular beam epitaxy, chemical vapor deposition, and laser ablation (top-bottom approaches) (33). The physio-chemical route provides the ability to make morphological changes in size and geometry of the resulting nanoparticles (34). Recently, a green process of ZnNPs synthesis has emerged as an alternative to the conventional method. It involves unicellular and multicellular biological entities, such as yeasts (35), bacteria (36), fungi (37, 38), viruses (39) algae (40) or various plant extracts (41). The main advantage is that green synthesis techniques embrace the use of ecofriendly and safe solvents such as water or natural extracts. One critical aspect demanding further investigation is the feasibility of increasing the production yield to the industrial scale (34). Despite this novel green synthesis approach, there are still some disadvantages of large-scale production and usage of ZnNPs (compared to ZnO) in the agriculture sector, namely: high capital cost, high energy requirements, and the use of hazardous chemicals. These consequences can cause unfavorable secondary pollution of the environment. Therefore, more exploration is needed for developing cleaner, environmentally safe, and economically accessible biocompatible alternatives for NPs synthesis.

ZnNP application in agriculture must be approached with some caution because some studies have pointed out the toxicity of ZnNPs (42). Concerning the fact that ZnNPs are prospective antimicrobials, their toxicity for the organism must be taken into account. It has been observed, ZnNPs (39 nm) showed dose-dependent toxicity. The dose of 100 mg/kg bw showed in the rat model, caused the most significant changes in liver enzymes, antioxidant system, and histopathological structure compared to 100 mg/kg/bw ZnO which had no significant toxic effects at this level (43). The mechanism of ZnNPs toxicity is similar to their antimicrobial effect. In addition to its basic nature, Zn shows significant toxic potential if levels in the biological system exceed a certain threshold. In both cases, the mode of action is based on the binding of Zn to peptides. Zn toxicity is mediated by oxidative stress, lipid peroxidation, cell membrane damage, and oxidative DNA damage (44). However, information concerning Zn toxicity relative to particle size is contradictory. Warheit et al. (45) did not observe any difference between large and small NPs, whereas (46) detected DNA damage significantly increased after cell exposure to larger NPs compared with smaller NPs. These observations encourage caution in their use. The size of ZnNPs should be characterized exactly. Depending on the size, structure, and composition, the ingestion of NPs can cause toxicity due to numerous physiological mechanisms. The small size of nanoparticles means they have a high specific surface area, which offers a large area for adsorption of any surface-active components in the GIT (47).

### Fate of ZnNPs in the GIT

**Figure 1:**
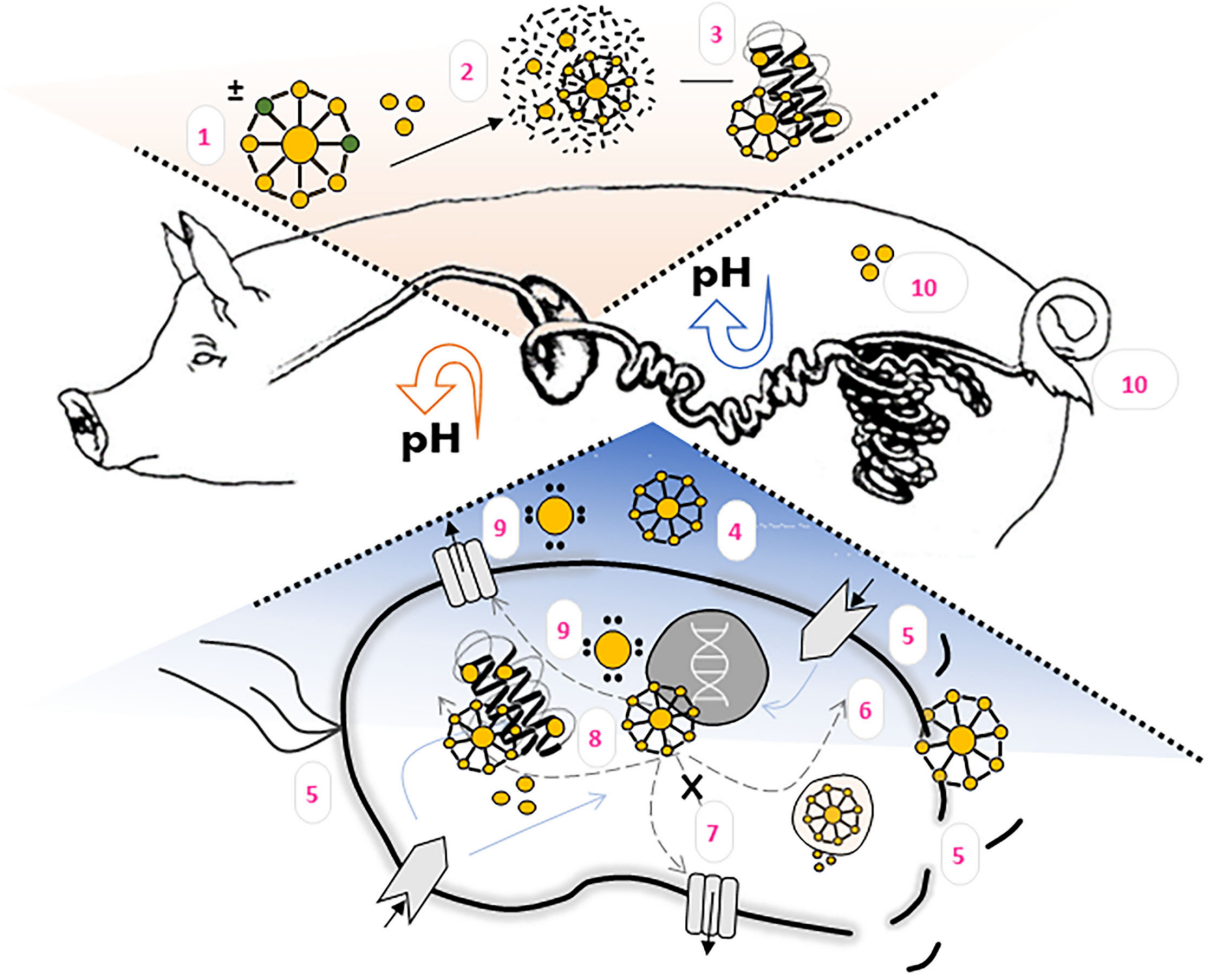
ZnNPs' fate and proposed mechanism of action in the pigs organism. (1) ZnNPs morphology, size, surface charge and other physico-chemical properties influence its bioavailability, antimicrobial activity, adhesion on epithelial cells, metabolization and (2) Zn^2+^ ions release. Both, ZnNPs and Zn^2+^ can interact with feed matrix as well as (3) biomolecules. In intestines, where the pH is increasing, ZnNPs or released Zn^2+^ ions can disrupt bacterial biofilm (4), influence surface area components, or disrupt bacterial cell membrane (5). ZnNPs are able to penetrate inside bacterial cell (6), where interaction with signaling pahways (7) or bacterial components (8) can occurr. The formation of ROS (9) has been observed either inside or outside bacterial cell. Zn bioaccumulation and excrection should be taken into account when evaluating the effectiveness of the particles (10).

Inorganic nanoparticles are not digested in the GIT, but some of them may be fully or partially dissolved as a result of alterations in pH or dilution (48). ZnNPs can be decomposed into Zn ions in acidic solutions or biological fluids (49). It has been shown that nanoparticle chemical composition plays a major role in determining their fate in the GIT. Thus, oral application of ZnNPs can be challenging due to poor drug solubility and stability in the GIT, the decreased bioavailability due to variable pH of the biological environment and protective mucus layer, or the presence of digestive enzymes (50). After oral ingestion, mechanical forces, biological fluids, and the changes of pH along the GIT can influence the biological action of ZnNPs in GIT (51). Furthermore, these actions can be affected by any binding with several macromolecules present in feed. It is believed that lipids, proteins, and polysaccharides which could interact with NPs are digested by proteases, lipases, and amylases in GIT (48). The proposed ZnNPs' mechanism of action in piglets' GIT is illustrated on Figure 1.

The biokinetics of ZnNPs was comprehensively reviewed by Choi and Choy (52). From this summarizing article clearly accrues, that the bioavailability of ZnNPs depends on their chemical form, size, and surface charge. ZnNPs smaller than 100 nm can pass through the stomach wall, more rapidly diffuse from the intestinal mucus and enter the cells of the intestinal lining into the blood system more quickly than ordinary minerals with larger particle size (50). It has been reported that the surface-charge effect plays a crucial role in ZnNPs bioavailability. Negatively charged ZnNPs are absorbed in larger amounts than positively charged ZnNPs (52). However, the mechanism of this effect is still unclear. It is known that intestinal epithelial cells possess a negatively charged cell surface which provides electrostatic interaction with positively charged compounds (53). Penetration of NPs under normal healthy conditions cannot cross the blood vessel endothelium into the target tissue. But in certain pathological conditions, e.g., inflammation, endothelial cells lose cellular integrity due to the activation of pro-inflammatory cytokines and the distance between endothelial cells is increased. As a result, ZnNPs can extravasate from the vasculature to the diseased site through the abnormal endothelial gap (50). ZnNPs tend to accumulate in size-dependent manner in the liver, kidneys, lungs, and other internal organs (54). Additionally, the excretion of ZnNPs is controlled by nanoparticle size. The smallest ZnNPs could be eliminated *via* renal clearance, whereas when the NPs size increases, they are eliminated via fecal excretion (55).

### ZnNPs Antibacterial Mechanism of Action

ZnNPs belongs to the most studied group of inorganic nanoparticles in the field of microbiology, as many researchers want to know more about their antimicrobial effect, with a rapidly increasing trend in this research since 2012. During this time, the antimicrobial mechanisms of action on ZnNPs against various microorganisms *in vitro* and *in vivo* have been more and more scrutinized and spotlighted. Current knowledge proposes three mechanisms of the ZnNPs antimicrobial effect: (i) production of ROS; (ii) disruption of bacterial cell wall integrity; (iii) release of Zn^2+^ ions which leads to interaction with biomolecules and vital functions of bacteria (56, 57). By any of these means, it has been found that G+ bacteria are more susceptible to ZnNPs compared to G- bacteria either by the disruption of bacterial integrity, damage by ROS or downregulation the transcription of oxidative stress-resistance genes.

Due to the semiconductor and electric properties of ZnNPs, they can produce superoxide anions (O^2−^, HO^−^ and H_2_O_2_) which are able to interact with anionic cell walls. The mechanisms of cellular toxicity that elevate ROS production, exceeding the capacity of antioxidant defense systems, cause cells to enter a state of oxidative stress. The result of this is the damage of cellular components such as lipids, proteins, and DNA (58). Fatty acid oxidation leads to the generation of lipid peroxides that initiate a chain reaction resulting in disruption of plasma and organelle membranes, leading to cell death (59). The study *in vitro* (60) had indicated that various environmental conditions can cause differences in ZnNPs antimicrobial activity against *E. coli* and *S. aureus*. Environmental temperature also influences antibacterial activity due to its effect on the generation rate of ROS. When ZnNPs are stimulated by temperature, electrons are captured at the active sites. Afterward, the electrons interact with oxygen to produce ROS, thereby enhancing the antimicrobial effectiveness of ZnNPs.

Another mechanism comes from their ability to pass through the cell wall *via* the bacterial peptidoglycan fence due to their small size (5). The various effect on ZnNPs was observed in G+ and G- bacteria which is based on differences in bacterial cell structure. The bacterial cell wall is responsible for the osmotic pressure of the cytoplasm as well as the characteristic cell shape. This in turn it means that the construction of bacterial walls is one of the factors in the effectiveness of antibiotics. G+ bacteria have one cytoplasmic membrane with a multilayer of peptidoglycan polymer and a thicker cell wall (20–80 nm) (61). In contrast, the G- bacteria wall is composed of two cell membranes, an outer membrane and a plasma membrane with a thin layer of peptidoglycan (10) with a thickness of 7–8 nm. This points to bacteria being highly susceptible to damage because the NPs can readily pass through the peptidoglycan cell due to their high durability (62). Overall, the antimicrobial properties of ZnNPs are based on the electrostatic interactions between NPs and cell surface, in addition to aggregation and damage inside the cell (62).

In neutral or alkaline pH the ZnNPs remain intact. In acidic media, ZnNPs are able to release Zn^2+^ ions which can interact with several biomolecules such as proteins and carbohydrates (63). The partial dissolution of ZnNPs can occur in the stomach where the pH range is 2–3. Moreover, when they penetrate a bacterial cell, lysosome is formed, and the rapid decrease of pH can cause Zn^2+^ to release. Inside a bacterial cell, released Zn can affect internal metabolism and signaling pathways, which can lead to death. Al-Shabib et al. (64) reported that the released Zn^2+^ ions concentration of ZnNPs in the simulated gastric fluid was six-times higher than that of an unprotected ZnO (65) which led to higher toxicity for *E. coli in vitro*. Similar effects were shown in the study of Sirelkhatim et al. (10), where the antimicrobial activity of ZnNPs had been partly attributed to the ability of Zn penetration into the microbial cells and generation of ROS that damage key cellular components.

In terms of the Zn ions' release from NPs, there is less concrete knowledge about the direct effect of ZnNPs on bacterial surface components such as virulence factors (adhesins, invasins, impedins, modulins, agresins). Only a minimal number of studies were found by searching the literature. For example, it was observed the ZnNPs suppress biofilms formation in *Chromobacterium violaceum, E. coli* PAO1, *Listeria monocytogenes, Streptococcus pneumoniae*, and *S. aureus* in the dose-dependent manner (64, 66, 67). Green synthetized ZnO and xanthan gum nanocomposite showed inhibition effect to violacein (61%) chitinase (70%) in *C. violaceum* and prodigiosin (71%) and protease (72%) in *S. marcescens* at 128 mu g/mL concentration (68). In the term of *E. coli*, the ZnNPs showed efficiency as an anti-inflammatory agent and suppressed multidrug resistance producing virulence and resistance genes in *E. coli* isolated from chicken meat (69).

However, as with other antimicrobial compounds, concerns about bacterial resistance have been raised. Some studies reported that bacterial tolerance to NPs may be due to electrostatic repulsion, mutations, biofilm adaptation, efflux pumps, and expression of extracellular matrices in the bacterial system (70–72). At the same time, there is a possibility of deleterious effects on animal and human health due to microbiota disturbance influenced by external factors (such as diet) after exposure to toxic feed additives, antibiotics or drug use, infections, and the environment (73). In another study, it has been reported that NPs mechanisms of action did not correspond to the most of antibiotic effects and act in several ways simultaneously, thus NPs are a promising tool to bypass the bacteria's resistance mechanisms (74).

### Influence of ZnNPs Structure on Antimicrobial Effect

The following factors play pivotal roles in ZnNPs' mechanism of action: its morphology, concentration, size, surface charge, modification or doping. All of these factors influence not only antimicrobial properties, but also bioavailability, biodistribution, elimination, reaction of immune system, interaction with host cells or food and feed matrix. It must be remembered, however, that *in vitro* observations can never predict the behavior of particles *in vivo* and their impact on the host organism or its microbiome.

For the purpose of antimicrobial effect, there is a need to transport ZnNPs in high concentration, without any chemical changes which could influence their behavior. Simultaneously, the toxic effect and systemic damage to the organism is obviously not desirable (75). Many studies have been published which agree that the antimicrobial effect depends on their size. The smaller the NPs, the higher the antimicrobial effect. Since the most cited research has focused on clarification of size-dependent bacterial growth under ZnNPs treatment, there have been introduced several studies which shown a promising contribution in antimicrobial activity using plant extracts to synthetize ZnNPs. A promising aspect is the presence of phytoactive compounds in the NPs structure which can promote its additional properties (76). However, few studies compared these two means of synthesis, and some of them showed evidence that green synthesized ZnNPs even had lower antimicrobial effect (77, 78). Moreover, disadvantages of the green synthesis include poor control of NPs formation, difficulty in large-scale production and differences in compound composition in plant extracts (79). Similar discussion has been held regarding to efficiency of modified and doped ZnNPs. The benefits of the modification are that it protects them from the acidic environment in the stomach, prevents aggregation and/or ensures invisibility to the immune system and prevents protein corona formation (80). Also, doping of ZnNPs by metal ions such as iron shown improving effect on ZnNPs antimicrobial activity (81).

## Weaning of Piglets and Diarrhea Occurrence

In the modern pig industry, weaning usually occurs between 21 and 28 days after birth (82). Several aspects contribute to the consideration of weaning as a stressful period for piglets, as they experience dramatic physiological, environmental, and social changes in the weaning transition (82). These changes can lead to pigs' diarrhea which leads to increased mortality in neonatal and young piglets. Diarrheal diseases may be caused solely by the presence of pathogenic microorganisms, but there is often a multifactorial cause (83). During weaning the nutrient intake is decreased significantly in the first days after the stressful change of place and nutrition from milk to carbohydrates. The lack of nutrient intake in this time leads to reduced proliferation of epithelial cells and increased production of the intestinal mucosa. This is usually followed by gut atrophy and loss of mucosal proteins (84) as well as periods of reduced growth rate, starvation, and severe anorexia after weaning (85). The negative effect of weaning can also occur in the stomach, where HCl is not sufficient to stimulate proteolysis and protection against pathogens and nutrients to stimulate the secretion of growth factors with intestinal trophic effect (86). These stressful events can lead to health problems and poor growth performance, therefore, increasing the possibility of post-weaning diarrhea due to microbiome disruption.

Piglets are also sensitive to changes in diet composition. It was observed that proteins and amino acids are directly related to the incidence of diarrhea in piglets. These undigested N-substances become a breeding ground for pathogenic microorganisms. It is generally known that the reduction of protein intake by about 1% (below 20% of total N-substances) leads to a decrease in the incidence of post-weaning diarrhea by 20–30%. The disadvantage is that the performance of pigs may be reduced, although the current genetic pool of pigs is able to catch up with this growth depression in the following days and weeks (87). On the contrary, some amino acids have been considered as beneficial for maintaining gut health, particularly from a morphological and microbiota perspective. Arginine, glutamine, methionin and threonine help alleviate post-weaning stress in young pigs by improving immunological functions, anti-inflammatory ability or antioxidant capacity (88). At the polysaccharide level, the increased intake of dietary fiber leads to higher incidence of diarrhea. However, fermentation of dietary fiber in the large intestine was shown to improve gut maturation by providing short-chain fatty acids to colonic mucosa and inhibit the adhesion of pathogenic bacteria (89). Research has also shown the benefits of resistant starch which is associated with butyrate-producing *Faecalibacterium prausnitzii* and the supression of pathogenic bacteria (88). During this period, piglets are more prone to the development of microbial infections, which is the second most common cause in addition to dietary changes.

### Post-Weaning Diarrhea in Piglets on the Microbiome Level

The colonization of the GIT of piglets occurs immediately after delivery and changes dynamically during the suckling, or weaning phase. Immediately after the birth, pigs' intestines are colonized by *Clostridiaceae* and *Enterobacteriaceae* families (90). The diversity of the microbial communities in the fecal samples further increase during weaning (91). In adult pigs, the number of microorganisms stabilizes at 1 × 10^10^-1 × 10^11^ (91, 92). Meta-analysis of 16S rRNA amplicon sequence data showed the pig-specific species are *Lactobacillus, Streptococcus, Clostridium, Desulfovibrio, Enterococcus, Fusobacterium*. Also, several new genera have been described within the same study (93). The microbial diversity depends on various internal and external factors, such as are breed, sex, but also environment, feed composition, or other factors, such as stress (30, 93). Inter-farm variability of the microbial profile of piglets has shown the variability of relative abundances of *Christensenellaceae* and *Lactobacillus* in suckling piglets. However, the most abundant microbial families did not differ in weaned piglets (94). It has been shown, the microbial colonization has impact on pigs' health and performance (94). Genome-based mapping of the microbial community revealed the importance of specialized bacterial metabolites for microbe-microbe and microbe-host interactions (95). During the suckling phase, the dominating pathways are lipid metabolism pathways (such as lipid biosynthesis proteins), energy metabolism pathways (such as carbon fixation) and carbohydrate pathways (such as the citrate cycle). The nicotinate and nicotinamide metabolism which is related to the metabolism of cofactors and vitamins increased in weaned piglets compared to lactation. Moreover, weaned piglets showed a higher proportion of pathways involved in bacterial chemotaxis, motility proteins, flagellar assembly, lipopolysaccharide biosynthesis and sporulation pathway (96). Predictably, the stress response (oxidative stress and heat shock) gene families have been significantly enriched in the weaned piglets (91). The interaction network of pigs' microbiota have been recently reviewed and could be concluded, that maintaining a balance of interactions and synergistic effects of the intestinal microflora, whether diet, environment and proper welfare, has a significant effect on pig performance (97–99).

At the phylum level, the most abundant are *Bacteroidetes, Firmicutes, Proteobacteria, Spirochetes* in fecal microbiota of weanling piglets, whereas *Fusobacteria* is significantly reduced. At the genus level, the microbiome is dominated by *Prevotella* and *Bacteroides* from *Bacteroidetes* phyla. A significant increase of *p-75-a5, Roseburia, Ruminococcus, Coprococcus, Dorea* and *Lachospira* have been observed simultaneously with significant decrease of *Bacteroides, Fusobacterium, Lactobacillus* and *Megashare* (95). On the contrary, Guevarra et al. showed the populations of *Prevotellaceae* and *Lactobacillaceae* significantly increased in weaned piglets (91). Another study also mentions an increase of genera *Veillonellaceae* and *Succinovibrionaceae* (94). The interrelationships between microorganisms affect the metabolic and trophic functions of the microbiome and may suggest a susceptibility to certain diseases, including post-weaning diarrhea. For example, subjects with a high abundance of *Prevotella* usually have a lower prevalence of *Bacteroides* which have immunomodulatory effects (100, 101). Possible explanation could be that Prevotella produce anti-inflammatory short fatty acids which help to modulate the immune system (102). In addition, Prevotella-driven enterotype has shown a positive influence of feed intake, weight gain, and results in the decrease of diarrhea incidence (103). Comprehensive research of weaning piglets' microbiota have been conducted by Dong et al. They published a collection included 266 cultured genomes and 482 meta-genome-assembled genomes that were clustered to 428 species cross 1% phyla. They found that *Limosilactobacillus reuteri* was the most abundant species and represented 18% of the taxa isolated and half of these were *Lactobacillus* (104).

The main bacterial strains that primarily cause diarrhea in weaned pigs are considered pathogenic *E. coli* strains, *Campylobacter* spp. *Clostridium perfringens* and *Salmonella* spp. (105). The onset of post-weaning diarrhea due to *E. coli* is primarily caused by toxin secretion. The heat-labile (LT) and two heat-stable (STa and STb) adhesin strains K88 can secrete into target cells of the intestinal epithelium causing diarrhea. Even though they trigger different pathways, all toxins disrupt tight connections and activate the massive luminal secretion of electrolytes and water (106, 107). Despite activating various routes, all the toxins activate pro-inflammatory cytokines, affect tight junction functions, stimulate enormous luminal electrolytes, water secretions, and eventually cause diarrhea (32).

Several studies have explored the importance of the relationship between microbial composition and the occurrence of post-weaning diarrhea. It has been established that the beneficial microbiota are helpful in diarrhea prevention due to immunomodulation and interventions in defensive metabolic pathways (27). Results from recent research (88) which analyzed fecal microbiota in diarrheic piglets revealed that diarrhea was associated with the increase of *Prevotella, Sutterella Campylobacter*, and *Fusobacterium* and the decrease of *Prevotellacea, Lachnospiraceae, Ruminococcaceae, Fusobacteriaceae* and *Lactobacillaceae* (88, 91). In the case of the *Fusobacterium*, the higher expression of inflammatory factors that inhibits T-cell responses has been observed (108, 109). Intestinal inflammation contributes to creating such an environment, which is convenient for the growth of several diarrhea-connected pathogens. *Salmonella enterica* and enterotoxigenic *E. coli* belong to the *Enterobacteriaceae* family which prevail at a time when the population of *Bifidobacterium, Lactobacillus* is declining whereas *Citrobacter, Clostridium, Rumonococcus* and *Diallister* is increasing (110). The microbial diversity and evenness in diarrheal pigs differed over time according to their subsequent susceptibility to post-weaning diarrhea. The taxonomic composition showed the highest differences in the phyla *Firmicutes* and *Bacteriodetes*. The abundance of *Lachnospiraceae, Ruminococcaceae* and *Prevotellaceae* was increased, whereas the presence of *Fusobacteriaceae* and *Corynebacteriaceae* was decreased in healthy pigs compared to pigs with diarrhea (111). In a newly released study, the microbial colonization of healthy and pigs with diarrhea differs on the intestinal segments level. The most abundant were *Lactobacillus* genus (38%), *Escherichia*-*Shigella* and *Enterococcus* (11%), *Bacteroides* (9%), *Fusobacterium* (8%), *Streptococcus* and *Prevotella* (3%), *Blautia* and *Clostridium* (1%). The significant decrease was observed in *Lactobacillus, Bacteroides* in pigs with diarrhea compared to healthy pigs. *Escherichia Shigella, Streptococcus, Sphaerochaeta* and *Enterococcus* were increased in pigs with diarrhea compared to the healthy group. Surprisingly, the bacterial diversity was higher in the healthy pigs compared to pigs with diarrhea among all intestinal segments (illeum, caecum, colon and rectum) (112).

Nevertheless, it should be noted that although some studies differ in the microbial composition in the GIT of piglets, the susceptibility of piglets to diarrhea is not only based on the presence of “good” and “bad” bacteria. Some pathogenic microflora belong to the group of opportunistic pathogens and could co-exist together without any negative impact. It is necessary to take into the account the overall health as well as feed composition and welfare of animals (99).

### The Microbiome and the Effect of ZnO and ZnNPs

Currently, there is a transition period between the EU ban of Zn medication doses in pig breeding and the employment of new approaches for reducing weaning pigs' diarrhea. Thus, there are some new studies emerging which are focused on the examination of the effects of both ZnO and ZnNPs on the intestinal microbiome of weaned piglets. In addition, methods such as 16S rRNA sequencing, next-generation sequencing and bioinformatics are more available among research teams. To date, there are several studies that are clarifying changes at a microbiome level in weaned piglets treated with medication doses of Zn. ZnO has been considered as effective against the abundance of opportunistic pathogens such as *Campylobacterales, Enterobacteriaceae* and *Escherichia* (5, 113, 114). Moreover, the abundance of beneficial bacteria such as *Lactobacillus* genera has been found in ZnO treated piglets (114, 115). Previous studies have shown the Zn is a significant factor in modulating the host microbiota and its biodiversity in dose dependent manner. Physiological doses of Zn could enhance species capable to improving gut wall integrity and immunomodulation. On the contrary, Zn overexposure could reduce bacterial diversity and lead to systemic inflammation (116). Based on analysis of growing on selective media, it was evident that 2,000 mg/L of ZnO caused the decrease of total anaerobes in stomach and small intestine, but not in caecum and colon. Lactobacilli were decreased in all parts of GIT. Coliforms were increased in caecum and colon, whereas enterococci were increased in all parts of GIT. 16S rRNA gene sequencing showed the decrease of *L. amylovorus, L. reuteri* and *S. alactolyticus* in the group treated by high doses of ZnO (2,000 mg/L) compared to the group with low dose of ZnO (100 mg/L) (117). A similar study (118), which compared porcine microbiome under 40 and 2,500 mg/L ZnO treatment, showed clear differences at genus and species level using metagenomic sequencing of colon microbiota. This study has shown the higher increases were in the genus of *Prevotella, Roseburia, Bacteroides, Bacillus* and the higher decreases in the genus of *Lactobacillus, Megaspharea* and *Alisteps*, whereas *Clostridium, Eubacterium, Dorea, Streptococcus* and *Bifidobacterium* remained unchanged The *Prevotella*/*Bacteroidetes* ratio in the gut microbiota has been shown to predict body weight in humans, in *Prevotella* pigs the rich enterotype has a beneficial effect on inflammation reduction in finish pigs due to the fact that representatives of the genus *Prevotella* use carbohydrates and produce anti-inflammatory short-chain fatty acids (102). The *Prevotella*-controlled enterotype showed a positive relationship, including feed intake and efficiency, weight gain, and prevention of diarrhea. This suggests that prevotella is important for mediating the growth performance and resistance of piglets to disease (103).

One of the first comparative studies between ZnO and ZnNPs in weaning piglets was presented by Oh et al. (119). This comprehensive study found that fecal score among various Zn forms (2,000 mg/kg ZnO, chelate-ZnO and 200 mg/kg ZnNPs) differed among phases (0–7, 7–14 days), but in the overall experimental period the control group showed lower fecal score compared to the treatment. The supplementation of various forms of Zn showed the significant increase in the genera *Prevotella, Succinivibrio* and *Lactobacillus*. Both species, *Prevotella* and *Succinivibrio* are related to the transition from milk to diet for weaning piglets, where *Prevotella* degrades polysaccharides in plant cell walls to short-chain fatty acids while *Succinivibrio* has cellulolytic activity (120–122). The specific differences between the bacterial abundance in ZnO and ZnNPs treated groups as well as the comparison among other studies can be found in Table 1. This study is in agreement with previous research where ZnNPs (600 mg/kg) increased bacterial richness and diversity in ileum and increased abundance of *Streptococcus* and *Lactobacillus* species in ileum, whereas the occurrence of *Oscillospira* and *Prevotella* decreased in colon. On the contrary, the dose of 600 mg/kg of ZnNPs was more effective in preventing pigs' diarrhea compared to the results of Oh et al. (123). Proposed mechanism of protective action of ZnNPs was explained by determination of mRNA expression of Cu-Zn SOD, GPX1, ZO-1 and Occludin in the jejunal tissue. However, an expression of mRNA was lower in ZnNPs-treated group compared to the ZnO group. Moreover, the study brings support to the evidence that in ZnO, free Zn is released more easily when compared to ZnNPs. This is probably due to the higher expression of metallothionein, which expression depends on Zn concentration in the body (127).

**Table 1:** Comparison of ZnO and ZnNP effects on pig microbiomes.

**MTD**	**SPL**	**Zn**	**Dose**	**Increased MO populations**	**Decreased MO populations**	**Remained MO populations**	**Associated findings**	**Reference**
NGS	F.	ZnO	2,500	Genus level: Prevotella, Roseburia, Bacteroides, Bacillus, Treponema, Escherichia, Faecalibacterium, Coprococcus, Shigella, Blautia, Acetivibrio; Dominant genera: Bacteroidetes, Actinobacteria, Firmicutes	Lactobacillus, Megaspharea, Alistipes, Dialister, Ruminococcus, Mitsuokella, Oscillibacter, Mycoplasma, Acidaminococcus	Clostridium, Eubacterium, Dorea, Streptococcus, Bifidobacterium	ZnO 2,500 mg/kg group compared to CTRL: sporadic occurrence of diarrhea; ↑ ADG in; ↑ expression of ZnT1, MT1A, and MT2B genes; decreased MO diversity; ↓ SCFA level, NH4, ↑ AR genes: aph(3”)-Ib, blaROB, pat(A), lnu(C), arnA	(118)
16S	I.	ZnO	2,000	Firmicutes (Streptococcaceae, Clostridiaceae, Bacillaceae), Proteobacteria (Halomonadaceae)	Firmicutes (Lactobacillaceae)		↑ diarrhea incidence in ZnNPs group compared to ZnO but ↓ compared to CTRL; ↑ expression of Cu-Zn SOD, GPX1, ZO-1, MT and Occludin in jejunum in ZnNPs group compared to ZnO; ↑ expression of CDK4 mRNA, ↓ Caspase3 and inflammatory markers mRNA in ZnNPs; group; ↑ richness and diversity of MO community in ZnNPs group	(8)
		ZnNPs (25 nm)	600	Firmicutes (Streptococcaceae, Clostridiaceae, Bacillaceae), Proteobacteria (Halomonadaceae)	Firmicutes (Lactobacillaceae)			
	Ce.	ZnO	2,000	Firmicutes (Streptococcaceae, Clostridiaceae), Actinobacteria (Coriobacteriaceae)	Firmicutes (Lachnospiraceae, Ruminococcaceae)			
		ZnNP (25 nm)	600	Firmicutes (Lactobacillaceae, Streptococcaceae, Clostridiaceae), Actinobacteria (Coriobacteriaceae)	Firmicutes (Lachnospiraceae, Erysipelotrichaceae, Ruminococcaceae)			
	Co.	ZnO	2,000	Actinobacteria (Coriobacteriaceae), Firmicutes (Streptococcaceae, Lactobacillaceae)	Bacteroidetes (Prevotellaceae, S24-7, Ruminococcaceae)			
		ZnNP (25 nm)	600	Actinobacteria (Coriobacteriaceae), Firmicutes (Streptococcaceae, Lactobacillaceae)	Bacteroidetes (Prevotellaceae, S24-7), Firmicutes (Veillonellaceae, Erysipelotrichaceae, Lachnospiraceae, Ruminococcaceae)			
CFU	I.	ZnO	2,500	Total anaerobic bacteria, *Lactobacillus* spp.	*Clostridium* spp., Coliforms		ZnNPs group: ↑ ADG; ↑ CP digestibility; ↑ Zn digestibility; quadratic ↑ in villus height and crypt depth ratio in duodenum, jejunum and ieum	(123)
		HME-ZnO	500	*Lactobacillus* spp.	*Clostridium* spp., Coliforms			
			1,000	Total anaerobic bacteria, *Lactobacillus* spp.	*Clostridium* spp., Coliforms			
			2,500	*Lactobacillus* spp.	*Clostridium* spp., Coliforms			
	Ce.	ZnO	2,500	Total anaerobic bacteria, *Lactobacillus* spp.	*Clostridium* spp., Coliforms			
		HME-ZnO	500	*Lactobacillus* spp.	Total anaerobic bacteria, *Clostridium* spp.			
			1,000	Total anaerobic bacteria, *Lactobacillus* spp.	*Clostridium* spp., Coliforms			
			2,500	Total anaerobic bacteria, *Lactobacillus* spp.	*Clostridium* spp., Coliforms			
	Co.	ZnO	2,500	Total anaerobic bacteria, *Lactobacillus* spp.	*Clostridium* spp., Coliforms			
		HME-ZnO	500		*Clostridium* spp., Coliforms			
			1,000	Total anaerobic bacteria, *Lactobacillus* spp.	*Clostridium* spp., Coliforms			
			2,500	Total anaerobic bacteria, *Lactobacillus* spp.	*Clostridium* spp., Coliforms			
qPCR	F.	ZnNP (100 nm)	20*	Bifidobacterium, Lactobacillus, Clostridium	Bacteroides, Enterococcus, Enterobacteriaceae		↑ body weight, ADG, ↓ feed conversion; ↑ ALP, ALT	(124)
16S	F.	ZnNP	200	Phylum level: Proteobacteria, Bacteroidetes, Spirochaetes. Genus level: Prevotella, Succinivibrio, Fibrobacter, Parabacteroides, Plaudibacter	Phylum level: Firmicutes, Lentisphaerae, Tenericutes. Genus level: Oscillospira, Roseburia, Treponema, Dorea, Lactobacillus, Campylobacter, Ruminococcus, Turicibacter		↑ diarrhea score in ZnO and ZnNPs; ↓ Zn excretion in ZnNPs group compared to ZnO; ↑ cytokine expression: GATA3+CD4+ T cells (Th2) and RORγt+CD4+ (Th17) T cells (gun lymph node) in ZnNPs group compared to ZnO and CTRL; no changes in cytokine expression (IL-8, IL-6, IFNγ, IL-1β, IL-10, IL-17A, and IL-22) among groups (gut lymph node); no changes of IL expression and TJ markers in intestinal tissues in ZnNPs group compared to CTRL	(119)
		ZnO	2,000	Phylum level: Bacteroidetes, Spirochaetes. Genus level: Prevotella, Treponema, Lactobacillus, Campylobacter, Ruminococcus, Parabacteroides, Paludibacter	Phylum level: Firmicutes, Proteobacteria, Lentisphaerae, Tenericutes. Genus level: Oscillospira, Succinivibrio, Roseburia, Dorea, Turicibacter, Fibrobacter			
16S	F.	ZnO	500	Genus level: Lactobacillus, Staphylococcus, Corynebacterium	Genus level: Escherichia, Streptococcus, Aerococcus,		↓ diarrhea occurrence in 1000 and 2000 mg/kg Zn; risk of diarrhea decreased ZnNPS A > ZnNPC C > ZnO; E. coli virulence genes STa, STb, Stx2, F4, F18 have occurred randomly but dramatically decreased at day 5 in treated groups (highest occurrence in ZnNPs A, lowest occurence in ZnNPs C and ZnO); ↑ level of GPx in ZnNPs A compared to other groups and CTRL; no differences in MDA levels were observed among all groups; ZnNPs A showed chronic enteritis with plaque focal atrophy and malabsorption syndrome; ZnNPs C showed mild inflammatory changes and higher expression of goblet cells	
			1,000	Genus level: Wautersiella, Staphylococcus	Genus level: Streptococcus, Aerococcus	Genus level: Escherichia, Lactobacillus		(125)
			2,000	Genus level: Enterococcus, Staphylococcus, Corynebacterium	Genus level: Escherichia, Streptococcus, Aerococcus, Lactobacillus			
		ZnNP A	500	Genus level: Escherichia, Pediococcus Enterococcus, Staphylococcus	Genus level: Streptococcus, Aerococcus, Lactobacillus			
			1,000	Genus level: Enterococcus, Staphylococcus	Genus level: Streptococcus, Aerococcus, Lactobacillus	Genus level: Escherichia		
			2,000	Genus level: Escherichia, Yersinia, Enterococcus	Genus level: Streptococcus, Aerococcus, Lactobacillus			
		ZnNP C	500	Genus level: Yersinia, Staphylococcus, Pediococcus, Lactococcus,	Genus level: Aerococcus, Streptococcus	Genus level: Escherichia, Streptococcus, Lactobacillus		
			1,000	Genus level: Enterococcus, Lactobacillus	Genus level: Streptococcus, Aerococcus	Genus level: Escherichia, Lactobacillus		
			2,000	Genus levelProvidencia, Enterococcus, Staphylococcus, Corynebacterium	Genus level: Streptococcus, Aerococcus	Genus level: Escherichia, Lactobacillus		
CFU	I.	ZnO	3,000		*Escherichia coli*	*Salmonella* spp., *Lactobacillus* spp., *Bacillus bifidus* spp.	↑ ADG in all treated groups; ↓ diarrhea occurrence in ZnO 3,000 mg/kg and ZnNPs 450 mg/kg; ↓ Zn excretion in ZnNPs group compared to ZnO and CTRL; pathological damage of villi in ZnNPs and ZnO groups compared to CTRL;	(115)
		ZnNP (72 nm)	450		*Escherichia coli*	*Salmonella* spp., *Lactobacillus* spp., *Bacillus bifidus* spp.		
CFU	F.	ZnO	3,000			*Enterococcus* spp., *Lactobacilus* spp., *Escherichia coli*	↑ Zn fecal excretion in ZnNPs group in the dose dependent manner; ↑ Zn fecal excretion in ZnO group compared to ZnNPs; ↓ diarrhea occurrence in ZnO and ZnNPs group in dose-dependent manner (1-7 days) compared to CTRL;	(126)
		ZnNP (38 nm)	15–60		Linear decrease at the dose dependend manner: *Enterococcus* spp., *Lactobacilus* spp., *Escherichia coli*			

MTD, method; SPL, sampling; Zn, zinc form; MO, microbial; Ref, reference; NGS, new-generation sequencing; 16S, 16S rRNA sequencing; F, feces; I, illeum; Ce, cecum; Co, colon; HME-ZnO, hot melt extruded ZnNPs; CTRL, Control group; ADG, average daily intake; ZnT, zinc transporter; MT, metallothionein; SCFA, short chain fatty acids; AR, antibiotic resistance; CP, crude protein; TJ, tight junctions; Doses of ZnO and ZnNPs are expressed in mg/mL. Dose of ZnNPs marked with * consist of 5 mL of L. Plantarum suspension.

Recent studies (123) of ZnNPs modulation effect on pigs' microbiome is focused on the modified ZnNPs rather than crude ZnNPs. Hot melt extruded-based ZnNPs (HME-ZnO) differ from crude ZnNPs by coating ZnNPs with a polymer matrix which optimizes solubility and bioavailability of the carrier substance. It has been shown that the HME-ZnO and Zn diet decreased *Clostridium* spp. and coliforms in the ileum. *Lactobacillus* spp. were increased only in ZnO fed group. Cecal microbiota did not show differences among proposed treatments, only linear decrease of *Clostridium* spp. was observed with higher concentration of HME-ZnO. Dose-dependent decrease of *Clostridium* spp. was observed also in colon HME-ZnO-fed pigs. It should be noted, that the ZnO digestibility was lower compared to HME-ZnO which could lead to different effect on microbial composition. However, the effect on pig performance was the same for both high dose ZnO (2.5 mg/kg) and sub-pharmacological dose of HME-ZnO (500 and 1,000 mg/kg) (123).

Two types of ZnNPs based on phosphate were introduced as potential improving agents in weaning piglets' diarrhea. At the doses of 100, 500, and 2,000 mg/kg significant changes in the microbiota composition were observed compared to the control group. Significant shifts were observed in *Escherichia, Streptococcus* and *Aerococcus* genera at the doses of 500 mg/kg in ZnNPs A variant compared to the control group. Also, the abundance of *Providencia* and *Staphylococcus* were observed. Species such as *Enterococcus, Yersinia, Pediococcus* occurred in ZnNPs C variant compared to the control group. The higher dose of ZnNPs in ZnNPs A variant caused the increase of *Escherichia* compared to the ZnNPs C variant which was comparable with ZnO treated group. During the four experimental stages, there was noticeable reduction in bacterial diversity as well as the decrease of *Lactobacillus* occurrence. Also, the difference in the ZnNPs variant A and variant C suggested that the effect on ZnNPs strongly depends on its composition (125). Bacterial synergic effects in the intestinal microflora are well-known and could also play a role in enhancing the preventive effect against weaned piglets' diarrhea. In the study of Zheng et al. (124) the probiotic species *L. plantarum* have been introduced together with ZnNPs in pigs' diet after weaning. *L. plantarum* is also known as Zn tolerable bacteria (128) what was clearly visible from the results. The increased occurrence of *Bifidobacterium* and *Lactobacillus* and the decrease of *Enterococcus* and *Enterobacteriaceae* were predictors of improved pig's performance and reduced occurrence of diarrhea (129). In addition to *L. plantarum*, the microbiome of pigs comprises several bacterial strains that are tolerant to high doses of Zn. For instance, *Bacillus altitudinis* have been found in porcine microbiome (130). Moreover, its ability of Zn biosorption has been mentioned in the field of remediation purposes (130). Crespo-Piazuelo et al. found that supplementation of *B. altitudinis* spores improves porcine offspring growth performance (131). The most likely explanation is the stimulation of the immune system; however, the mechanism is not fully understood yet (93, 131).

The majority of previous studies on ZnNPs' efficiency focus mostly on the *in vitro* investigation of each specific microbial species. The advantage of these studies is the ability to monitor the effect of the composition and morphology of nanoparticles on the control of specific bacteria. However, *in vitro* studies mainly focus on important bacterial strains either as pathogens or prebiotic. Comparing the inhibitory concentrations from Table 2 (full version is available from repository: https://archive.org/details/osf-registrations-gqspd-v1), it is confirmed that ZnNPs act better on G+ bacteria, which are mainly the family *Lactobacillaceae, Enterococcaceae, Streptococcaceae*, although, inhibition activity is not negligible in the G- bacteria strains for family *Enterobacteriaceae* which are mostly pathogenic. A great deal of previous research into ZnNPs as antimicrobials has focused on the green synthesis. The purpose of green synthesis is, in addition to the ecological approach, also to use the potential of plant extracts, which have antibacterial properties, for the synergistic effect of ZnNPs. When compared to chemical route these initial observations suggest that green synthetized ZnNPs have less antimicrobial potential. However, the volume of this research annually increasing.

**Table 2:** Antimicrobial effect of ZnO nanoparticles tested *in vitro*.

**Size**	**S**	**Modification**	**Bacterial strain**	**G**	**Order**	**Pat**.	**MIC**	**IZ**	**Reference**
**nm**							**mg/mL**	**mm**	
57	GS	Punica granatum	*Salmonella typhimurium*	*G-*	*Enterobacterales*	P	6E-04		
57	GS	Punica granatum	*Listeria monocytogenes*	*G+*	*Bacillales*	P	7E-04		
57	GS	Punica granatum	*Enterococcus faecium*	*G+*	*Lactobacillales*	C	8E-04		(132)
57	GS	Punica granatum	*Aeromonas hydrophila*	*G-*	*Aeromonadales*	P	8E-04		
57	GS	Punica granatum	*Bacillus cereus*	*G+*	*Bacillales*	OP	9E-04		(133)
17	CR		*Salmonella typhimurium*	*G-*	*Enterobacterales*	P	0.002		
90	GS	Garcinia cambogia	*Staphylococcus aureus*	*G+*	*Bacillales*	OP	0.002	17	
90	GS	Garcinia cambogia	*Enterococcus faecalis*	*G+*	*Lactobacillales*	OP	0.002	18	
90	GS	Garcinia cambogia	*Proteus vulgaris*	*G-*	*Enterobacterales*	C	0.002	14	(134)
90	GS	Garcinia cambogia	*Pseudomonas aeruginosa*	*G-*	*Pseudomonadales*	P	0.002	15	
17	CR		*Escherichia coli*	*G-*	*Enterobacterales*	P	0.005		(133)
17	CR		*Staphylococcus aureus*	*G+*	*Bacillales*	OP	0.005		
275	GS	H. triquetrifolium	*Enterococcus faecalis*	*G+*	*Lactobacillales*	OP	0.005		(135)
17	CR		*Pseudomonas aeruginosa*	*G-*	*Pseudomonadales*	P	0.007		(133)
50	CO		*Streptococcus pyogenes*	*G+*	*Lactobacillales*	OP	0.01		(136)
20	CR		*Streptococcus mutans*	*G+*	*Lactobacillales*	OP	0.01	16	
20	CR		*Enterococcus faecalis*	*G+*	*Lactobacillales*	OP	0.01	14	
20	CR		*Lactobacillus fermentum*	*G+*	*Lactobacillales*	PR	0.01	10	
40	CR		*Streptococcus mutans*	*G+*	*Lactobacillales*	OP	0.01	14	(137)
40	CR		*Enterococcus faecalis*	*G+*	*Lactobacillales*	OP	0.01	13	
40	CR		*Lactobacillus fermentum*	*G+*	*Lactobacillales*	PR	0.01	9	
140	CR		*Streptococcus mutans*	*G+*	*Lactobacillales*	OP	0.01	12	
140	CR		*Enterococcus faecalis*	*G+*	*Lactobacillales*	OP	0.01	12	
140	CR		*Lactobacillus fermentum*	*G+*	*Lactobacillales*	PR	0.01	8	(138)
17	CO		*Escherichia coli*	*G-*	*Enterobacterales*	P	0.017		(133)
18	CR		*Enterococcus faecalis*	*G+*	*Lactobacillales*	OP	0.02	10	
18	CR		*Klebsiella pneumoniae*	*G-*	*Enterobacterales*	OP	0.02	10	
18	CR		*Escherichia coli*	*G-*	*Enterobacterales*	P	0.02	13	(138)
18	CR		*Staphylococcus aureus*	*G+*	*Bacillales*	OP	0.02	18	
275	GS	H. triquetrifolium	*Staphylococcus aureus*	*G+*	*Bacillales*	OP	0.02		
50	GS	Crocus sativus L.	*Salmonella typhimurium*	*G-*	*Enterobacterales*	P	0.02	12	
50	GS	Crocus sativus L.	*Listeria monocytogenes*	*G+*	*Bacillales*	P	0.02	ND	(139)
50	GS	Crocus sativus L.	*Enterococcus faecalis*	*G+*	*Lactobacillales*	OP	0.02	11	
29.5	BS	L. plantarum	*Staphylococcus aureus*	*G+*	*Bacillales*	OP	0.03		(140)
44.5	BS	Pseudomonas putida	*Bacillus cereus*	*G+*	*Bacillales*	OP	0.03		
17	CO		*Salmonella typhimurium*	*G-*	*Enterobacterales*	P	0.034		(141)
17	CO		*Pseudomonas aeruginosa*	*G-*	*Pseudomonadales*	P	0.041		(133)
17	CO		*Staphylococcus aureus*	*G+*	*Bacillales*	OP	0.047		
50	CR		*Bacillus subtilis*	*G+*	*Bacillales*	C	0.05		(142)
NA			*Lactobacillus fermentum*	*G+*	*Lactobacillales*	PR	0.05		
30	CO		*Lactobacillus plantarum*	*G+*	*Lactobacillales*	PR	0.05		(143)
33.5	CR		*Bacillus subtilis*	*G+*	*Bacillales*	C	0.05	6	
33.5	CR		*S.aureus (MRSA)*	*G+*	*Bacillales*	OP	0.05	10	
33.5	CR		*Staphylococcus aureus*	*G+*	*Bacillales*	OP	0.05	8	
33.5	CR		*Streptococcus mutans*	*G+*	*Lactobacillales*	OP	0.05	8	
33.5	CR		*Klebsiella pneumoniae*	*G-*	*Enterobacterales*	OP	0.05	10	
33.5	CR		*Escherichia coli*	*G-*	*Enterobacterales*	P	0.05	10	
26.5	CR	chitosan	*Bacillus subtilis*	*G+*	*Bacillales*	C	0.05	8	(144)
26.5	CR	chitosan	*S.aureus (MRSA)*	*G+*	*Bacillales*	OP	0.05	11	
26.5	CR	chitosan	*Staphylococcus aureus*	*G+*	*Bacillales*	OP	0.05	10	
26.5	CR	chitosan	*Streptococcus mutans*	*G+*	*Lactobacillales*	OP	0.05	10	
26.5	CR	chitosan	*Klebsiella pneumoniae*	*G-*	*Enterobacterales*	OP	0.05	14	
26.5	CR	chitosan	*Escherichia coli*	*G-*	*Enterobacterales*	P	0.05	13	
65	GS	Prosopis juliflora	*Escherichia coli*	*G-*	*Enterobacterales*	P	0.05	20	(145)
65	GS	Prosopis juliflora	*Bacillus subtilis*	*G+*	*Bacillales*	C	0.05	15	
65	GS	Prosopis juliflora	*Vibrio cholerae*	*G-*	*Vibrionales*	P	0.05	20	
29.6	BS	*L. plantarum*	*Escherichia coli*	*G-*	*Enterobacterales*	P	0.06		(140)
44.5	BS	*Pseudomonas putida*	*Pseudomonas otitidis*	*G-*	*Pseudomonadales*	C	0.06		
44.5	BS	*Pseudomonas putida*	*Enterococcus faecalis*	*G+*	*Lactobacillales*	OP	0.06		(141)
30	GS	*Psidium guajava*	*Escherichia coli (ETEC)*	*G-*	*Enterobacterales*	P	0.06	19	(146)
29.7	BS	*L. plantarum*	*Salmonella* spp.	*G-*	*Enterobacterales*	P	0.08		(140)
44.5	BS	*Pseudomonas putida*	*Pseudomonas oleovorans*	*G-*	*Pseudomonadales*	C	0.09		(141)
60	GS	*Pisonia grandis*	*Bacillus subtilis*	*G+*	*Bacillales*	C	0.1	12	
60	GS	*Pisonia grandis*	*Micrococcus luteus*	*G+*	*Actinomycetales*	C	0.1	16	(147)
60	GS	*Pisonia grandis*	*Salmonella paratyphi*	*G-*	*Enterobacterales*	P	0.1	14	
5	CR	Glucose-1-phosphate	*Prevotella intermedia*	*G-*	*Bacteroidales*	C	0.1		(148)
25	GS	*Ocimum basilicum*	*Staphylococcus sciuri*	*G+*	*Bacillales*	OP	0.1	14	
25	GS	*Ocimum basilicum*	*Salmonella enterica*	*G-*	*Enterobacterales*	P	0.1	13	(149)
25	GS	*Ocimum basilicum*	*Staphylococcus aureus*	*G+*	*Bacillales*	OP	0.1	18	
52	GS	*Cymodocea serrulata*	*Bacillus subtilis*	*G+*	*Bacillales*	C	0.1	18	(150)
52	GS	*Cymodocea serrulata*	*Escherichia coli*	*G-*	*Enterobacterales*	P	0.1	15	
52	GS	*Cymodocea serrulata*	*Klebsiella pneumoniae*	*G-*	*Enterobacterales*	OP	0.1	15	
52	GS	*Cymodocea serrulata*	*Shigella flexneri*	*G-*	*Enterobacterales*	P	0.1	14	
80	GS	*Punica granatum*	*Bacillus cereus*	*G+*	*Bacillales*	OP	0.1		(151)
80	GS	*Punica granatum*	*Bacillus licheniformis*	*G+*	*Bacillales*	PR	0.1		
80	GS	*Punica granatum*	*Escherichia coli*	*G-*	*Enterobacterales*	P	0.1		
8.34	GS	*Ephedra aphylla*	*Salmonella typhimurium*	*G-*	*Enterobacterales*	P	0.1	16	(152)
8.34	GS	*Ephedra aphylla*	*Pseudomonas aeruginosa*	*G-*	*Pseudomonadales*	P	0.1	17	
8.34	GS	*Ephedra aphylla*	*Klebsiella pneumoniae*	*G-*	*Enterobacterales*	OP	0.1	21	
8.34	GS	*Ephedra aphylla*	*Escherichia coli*	*G-*	*Enterobacterales*	P	0.1	20	
8.34	GS	*Ephedra aphylla*	*Staphylococcus epidermidis*	*G+*	*Bacillales*	OP	0.1	ND	
8.34	GS	*Ephedra aphylla*	*Bacillus cereus*	*G+*	*Bacillales*	OP	0.1	14	
8.34	GS	*Ephedra aphylla*	*Staphylococcus aureus*	*G+*	*Bacillales*	OP	0.1	19	
8.34	GS	*Ephedra aphylla*	*Listeria monocytogenes*	*G+*	*Bacillales*	P	0.1	20	
44.5	BS	*Pseudomonas putida*	*Acinetobacter baumannii*	*G-*	*Pseudomonadales*	OP	0.12		(141)
5	CR	Glucose-1-phosphate	*Fusobacterium nucleatum*	*G-*	*Fusobacteriales*	OP	0.2		(148, 153)
5	CR	PVP	*Salmonella enteritidis*	*G-*	*Enterobacterales*	P	0.28		(154)
5	CR	chitosan	*Staphylococcus aureus*	*G+*	*Bacillales*	OP	0.3		(155)
56	GS	PE	*Escherichia coli O157:H7*	*G-*	*Enterobacterales*	P	0.3		(153)
5	CR	PVP	*Listeria monocytogenes*	*G+*	*Bacillales*	P	0.3		(154)
56	GS	PE	*Listeria monocytogenes*	*G+*	*Bacillales*	P	0.35		(60)
4.45	CR		*Escherichia coli O157:H7*	*G-*	*Enterobacterales*	P	0.375		(154)
5	CR	Chitosan	*Lactobacillus fermentum*	*G+*	*Lactobacillales*	PR	0.4		(156)
30	CO		*Campylobacter jejuni*	*G-*	*Campylobacterales*	P	0.4		
30	CO		*Escherichia coli O157:H7*	*G-*	*Enterobacterales*	P	0.4		
30	CO		*Salmonella enterica*	*G-*	*Enterobacterales*	P	0.4		
60	CR	Aluminum	*Enterococcus hirae*	*G+*	*Lactobacillales*	P	0.5	10	(157)
60	CR	Aluminum	*Escherichia coli*	*G-*	*Enterobacterales*	P	0.5	10	
100	GS	Triphala extract	*Streptococcus mutans*	*G+*	*Lactobacillales*	OP	0.53	23	(158)
50	CR	Chitosan	*Enterococcus faecium*	*G+*	*Lactobacillales*	C	0.781		(159)
5	CR	Chitosan	*Escherichia coli*	*G-*	*Enterobacterales*	P	0.8		(154)
45	GS	O. americanum	*Salmonella paratyphi*	*G-*	*Enterobacterales*	P	1	24	(160)
45	GS	*O. americanum*	*Klebsiella pneumoniae*	*G-*	*Enterobacterales*	OP	1	27	
45	GS	*O. americanum*	*Clostridium perfringens*	*G+*	*Clostridiales*	P	1	30	
45	GS	*O. americanum*	*Bacillus cereus*	*G+*	*Bacillales*	OP	1	25	(161)
32	GS	*Lactobacillu* spp.	*Clostridium difficile*	*G+*	*Clostridiales*	P	1	21	
32	GS	*Lactobacillu* spp.	*Clostridium perfringens*	*G+*	*Clostridiales*	P	1	24	
32	GS	*Lactobacillu* spp.	*Escherichia coli*	*G-*	*Enterobacterales*	P	1	22	
32	GS	*Lactobacillu* spp.	*Salmonella typhimurium*	*G-*	*Enterobacterales*	P	1	20	
57	GS	*Punica granatum*	*Staphylococcus aureus*	*G+*	*Bacillales*	OP	1.25		(132)
57	GS	*Punica granatum*	*Klebsiella pneumoniae*	*G-*	*Enterobacterales*	OP	1.25		
57	GS	*Punica granatum*	*Enterococcus faecalis*	*G+*	*Lactobacillales*	OP	1.25		
57	GS	*Punica granatum*	*Escherichia coli*	*G-*	*Enterobacterales*	P	1.25		
57	GS	*Punica granatum*	*Moraxella catarrhalis*	*G-*	*Pseudomonadales*	OP	1.25		
12	CR	PEG	*Micrococcus luteus*	*G+*	*Actinomycetales*	C	1.25		(162)
4.45	CR		*Staphylococcus aureus*	*G+*	*Bacillales*	OP	1.5		(60)
97	CR	PVP	*Micrococcus luteus*	*G+*	*Actinomycetales*	C	2.5		(162)
5	CR	PVP	*Escherichia coli O157:H7*	*G-*	*Enterobacterales*	P	3.2		(153)
50	CR	Chitosan	*Escherichia coli*	*G-*	*Enterobacterales*	P	5		(159)
27.5	GS	*Brassica rapa*	*Micrococcus luteus*	*G+*	*Actinomycetales*	C	12.5	30	(163)
27.5	GS	*Brassica rapa*	*Klebsiella aerogenes*	*G-*	*Enterobacterales*	P	25	20	
25	GS	*Euphorbia hirta*	*Streptococcus mutans*	*G+*	*Lactobacillales*	OP	100	28	(164)
25	GS	*Euphorbia hirta*	*Clostridium absonum*	*G+*	*Clostridiales*	C	100	27	
25	GS	*Euphorbia hirta*	*Escherichia coli*	*G-*	*Enterobacterales*	P	100	24	
275	GS	*H. triquetrifolium*	*Escherichia coli*	*G-*	*Enterobacterales*	P	<0.1		
NA	GS	*H. triquetrifolium*	*Klebsiella pneumoniae*	*G-*	*Enterobacterales*	OP	<0.1		
275	GS	*H. triquetrifolium*	*Acinetobacter baumannii*	*G-*	*Pseudomonadales*	OP	<0.1		
5	CR	Glucose-1-phosphate	*Staphylococcus aureus*	*G+*	*Bacillales*	OP	<1		(148)
5	CR	Glucose-1-phosphate	*Lactobacillus paracasei*	*G+*	*Lactobacillales*	PR	<1		
275	GS	*H. triquetrifolium*	*Listeria ivanovii*	*G+*	*Bacillales*	P	ND		(164)
5	CR	Chitosan	*Enterococcus faecalis*	*G+*	*Lactobacillales*	OP	ND		(154)

*S, method of synthesis; G, Gram stain; Pat., Pathogenicity; MIC, minimal inhibitory concentration; IZ, inhibition zone; Ref., refference; GS, green synthesis; CR, chemical route synthesis; CO, commercial ZnNPs; BS, biosynthesis; PE, plant extract (Cinnamomum verum, Thymus vulgaris, Syzigium aromaticum)*.

*In vitro* effectiveness of ZnNPs against pathogenic bacterial species is summarized in Table 2, but they have also been described other reviews (10). Nevertheless, it should be considered that it is not the species/strains which are potentially harmful to the host. It would be better to prevent undesirable pathological conditions by reducing the high diversity in the coliform bacteria group rather than low coliform concentrations (87). For instance, ZnNPs may inactivate the growth of diarrhea-causing E. Coli, but as it has been recorded in several studies (8, 165), it may inactivate the growth of commensal beneficial microorganisms as well in the GIT, which could lead to dysbiosis of the intestinal microbiome. It has been shown that post-weaning diarrhea control attributed to ZnO might not be directly related to effects on microbiota, like reducing *Escherichia coli* count in the intestine, but it may be related to the prevention of bacterial adhesion of K88 strains and internalization to the intestinal epithelium, which reduces the deleterious effects of toxins produced by these pathogens (126). In the case of ZnNPs the anti-adhesive-effect have been studied with similar results. The proposed mechanism of action relies on the internationalization of ZnNPs to the hydrophilic bacterial cell wall surface or by inhibiting exopolysaccharides which are the main component of bacterial biofilm (166).

Several concerns have been raised about ZnNPs' effects against probiotic bacteria strains which are crucial for the healthy gut in weaning piglets. In Table 2 it can be seen that the inhibitory concentration for Lactobacillus spp. is ~0.01–0.1 mg/mL, which is slightly lower concentration than, for example, for pathogenic E. Coli strains (0.06–5 mg/mL). According to Li et al. the effect could be explained by the existence of free Zn^2+^ ions which are complexed with amino acids, resulting in the decrease of ZnNPs toxicity in *E. Coli* (44). What has been established is that some probiotic strains are tolerant for high levels of Zn or, interestingly, they may function as a biofactory for the synthesis of ZnNPs (128). Although the effect of ZnO has some similarities to the effects of ZnNPs, in particular the release of Zn^2+^ ions may vary (for further details see Section ZnNPs Antibacterial Mechanism of Action). The proteomic study showed a dramatic change of the metabolic pathways in Bacillus subtilis exposed to ZnNPs (0.017 mg/mL, 100 nm, Sigma Aldrich). Major changes have been observed in methylglyoxal and thiol metabolisms, oxidative stress and stringent responses (167). As some researches have suggested, the effect of NPs on microorganisms is dose- and time-dependent, so the impact of ZnNPs should be carefully considered to maintain balance in the environment (167). Other research has been focused on the interaction of ZnNPs with cell membrane of probiotic bacteria strains. It has been observed the commercial ZnNPs (77 nm, Alfa Aesar, 20 mM) did not damaged the cell wall of *Escherichia coli, Lactobacillus acidophilus*, nor *Bifidobacterium animalis*. Only slight cellular morphological changes were observed (168). To contradict this study, another research team observed that commercial ZnNPs (0.1 mg/mL, 80 nm, positive surface charge, Shanghai Macklin Biochemical) significantly changed the morphology of *Lactobacillus plantarum* and *Lactobacillus fermentum* and destroyed their cell membranes in the dose-dependent manner.

## Conclusions and Future Perspectives

The study of ZnNPs as a substitute for pharmacological doses of Zn in pig breeding is a very complex topic. Results of several research articles on ZnO's effects on microbiomes remain fairly inconsistent. However, ZnNPs are highly variable in their content and morphology, which affects their antimicrobial abilities and preventative effects against diarrhea in weaned piglets. Significant limitations of *in vivo* studies include a poor characterization of ZnNPs properties such as surface area, charge and size. It would be advisable to focus more on physico-chemical attributes of ZnNPs and interconnect these findings with information which already have been known on microbiomes. ZnNPs have substantial potential to replace pharmacological doses of Zn and aid the management of pig health in agricultural practices.

## Author Contributions

DB, AB, PH, and SS: conceptualization and writing—original draft preparation. SM, PH, SS, and JS: writing—review and editing. PH: supervision. All authors have read and agreed to the published version of the manuscript.

## Funding

This review article was supported by the Ministry of Agriculture of the Czech Republic (MZe) from the project QK1720349.

## Conflict of Interest

The authors declare that the research was conducted in the absence of any commercial or financial relationships that could be construed as a potential conflict of interest.

## Publisher's Note

All claims expressed in this article are solely those of the authors and do not necessarily represent those of their affiliated organizations, or those of the publisher, the editors and the reviewers. Any product that may be evaluated in this article, or claim that may be made by its manufacturer, is not guaranteed or endorsed by the publisher.
